# Dr. J. C. W. Lever

**Published:** 1859-03

**Authors:** 


DR. J. C. W. LEVER.—This promising physician died in London, on the 29th of December last, of disease of the heart, in the forty-eighth year of his age. As Physician-Accoucheur to Guy's Hospital, and Lecturer on Midwifery at that institution, to which he was appointed in 1845, he rapidly rose to eminence and usefulness in his peculiar department. He was the author of an excellent essay upon Organic Diseases of the Uterus, for which he obtained the Fothergillian gold medal in 1843, and contributed many valuable papers to *Guy's Hospital Reports*, the *London Lancet*, and other periodicals. His name is familiar to Philadelphians, as an honorary member of the College of Physicians.

